# O-linked N-acetylglucosamine transferase (OGT) regulates pancreatic α-cell function in mice

**DOI:** 10.1016/j.jbc.2021.100297

**Published:** 2021-01-16

**Authors:** Ahmad Essawy, Seokwon Jo, Megan Beetch, Amber Lockridge, Eric Gustafson, Emilyn U. Alejandro

**Affiliations:** Department of Integrative Biology and Physiology, University of Minnesota Medical School, Minneapolis, MN, USA

**Keywords:** diabetes, pancreatic alpha cell, O-linked N-acetylglucosamine (GlcNAc) transferase, O-GlcNAcylation, glucagon, apoptosis, proglucagon, carboxypeptidase E, ER, endoplasmic reticulum, GFP, green fluorescent protein, HBP, hexosamine biosynthetic pathway, IBP, Integrative Biology and Physiology, IPGTT, intraperitoneal glucose tolerance test, ITT, insulin tolerance test, OGT, O-GlcNAc transferase, PVN, periventricular nucleus

## Abstract

The nutrient sensor O-GlcNAc transferase (OGT) catalyzes posttranslational addition of O-GlcNAc onto target proteins, influencing signaling pathways in response to cellular nutrient levels. OGT is highly expressed in pancreatic glucagon-secreting cells (α-cells), which secrete glucagon in response to hypoglycemia. The objective of this study was to determine whether OGT is necessary for the regulation of α-cell mass and function *in vivo*. We utilized genetic manipulation to produce two α-cell specific OGT-knockout models: a constitutive glucagon-Cre (αOGT^KO^) and an inducible glucagon-Cre (i-αOGT^KO^), which effectively delete OGT in α-cells. Using approaches including immunoblotting, immunofluorescent imaging, and metabolic phenotyping *in vivo*, we provide the first insight on the role of O-GlcNAcylation in α-cell mass and function. αOGT^KO^ mice demonstrated normal glucose tolerance and insulin sensitivity but displayed significantly lower glucagon levels during both fed and fasted states. αOGT^KO^ mice exhibited significantly lower α-cell glucagon content and α-cell mass at 6 months of age. In fasting, αOGT^KO^ mice showed impaired pyruvate stimulated gluconeogenesis *in vivo* and reduced glucagon secretion *in vitro*. i-αOGT^KO^ mice showed similarly reduced blood glucagon levels, defective *in vitro* glucagon secretion, and normal α-cell mass. Interestingly, both αOGT^KO^ and i-αOGT^KO^ mice had no deficiency in maintaining blood glucose homeostasis under fed or fasting conditions, despite impairment in α-cell mass and function, and glucagon content. In conclusion, these studies provide a first look at the role of OGT signaling in the α-cell, its effect on α-cell mass, and its importance in regulating glucagon secretion in hypoglycemic conditions.

In the pancreas, glucose homeostasis is mainly regulated by the islets of Langerhans, which consist of different endocrine cells including the β-cells and α-cells (1). β-cells secrete insulin in response to high blood glucose. In contrast, α-cells secrete glucagon in response to low blood glucose levels. Together, these endocrine cells, secreting their counter regulatory hormones, work together to maintain blood glucose at a physiological level (2). For both type 1 (T1D) and type 2 (T2D) diabetes, a clear role for absolute deficiency or insufficiency in insulin has been established (3). In addition to altered insulin levels, dysregulation of glucagon levels in T1D and T2D contributes to the pathology of these diseases (4, 5). High glucagon levels in both T1D and T2D exacerbate hyperglycemia due to enhanced hepatic glucose output (6). While the cause and result of functional β-cell deficiency have been studied heavily in the field, the mechanisms and impact of α-cell failure are less known. Thus, a greater understanding of processes that regulate α-cell function may present new avenues for optimal glucose control in T1D or advanced therapy for T2D patients.

The hexosamine biosynthetic pathway (HBP) is a minor branch of glycolysis responsible for the production of the key substrate for protein glycosylation, UDP-GlcNAc (7). Posttranslational addition of UDP-GlcNAc (known as O-GlcNAcylation) is a dynamic and reversible process analogous to phosphorylation and has been shown to affect the function, stability, and subcellular localization of many proteins (8). O-GlcNAcylation is catalyzed solely by the enzyme O-linked GlcNAc transferase (OGT), by means of the addition of UDP-GlcNAc to the serine or threonine residues on nuclear and cytosolic proteins (9). Conversely, the O-GlcNAc is removed by the enzyme O-linked β-N-acetyl hexosaminidase (O-GlcNAcase or OGA) (10).

Approximately 3 to 5% of glucose entering the cell is shunted toward use by the HBP, and therefore, the degree of O-GlcNAcylation present in the cell is dependent on the amount of nutrients (glucose) present in the cellular environment (11). OGT is richly expressed in the pancreas (12, 13). At the protein level, OGT appears to be more abundantly expressed in islets compared with the acinar tissue (14). Within the islet, high OGT mRNA expression has been detected in both the α- and β-cells (12, 15). Recently, our group reported that O-GlcNAcylation is essential for cell survival and function. We reported that loss of O-GlcNAcylation in the β-cell leads to cell failure and diabetes in mice due to increased endoplasmic reticulum (ER) stress and apoptosis (14). Ablation of OGT in pancreatic progenitors also leads to an increased apoptosis (16). Independent of its role in cell survival, OGT also regulates insulin secretion at basal (14) and in obesity conditions in part through SERCA2 (17). Moreover, OGT regulates insulin processing *via* downstream target eIF4G1, a translation initiation factor (18). However, as a nutrient sensor protein that is highly expressed in glucagon-secreting cells, the role of OGT in α-cells has not been explored. We hypothesize that OGT plays a key role in the maintenance of α-cell mass and proper function of secreting glucagon in response to hypoglycemia.

It is unknown how nutrient-driven posttranslational O-GlcNAcylation of proteins impacts pancreas α-cell mass and function. In the currently study, through the characterization of mice lacking α-cell OGT, the only enzyme capable of adding O-GlcNAc modification onto proteins, we show that O-GlcNAcylation is necessary for the maintenance of α-cell mass and regulation of glucagon secretion.

## Results

### αOGT^KO^ mice show reduced OGT activity in glucagon-positive cells

**Figure 1:**
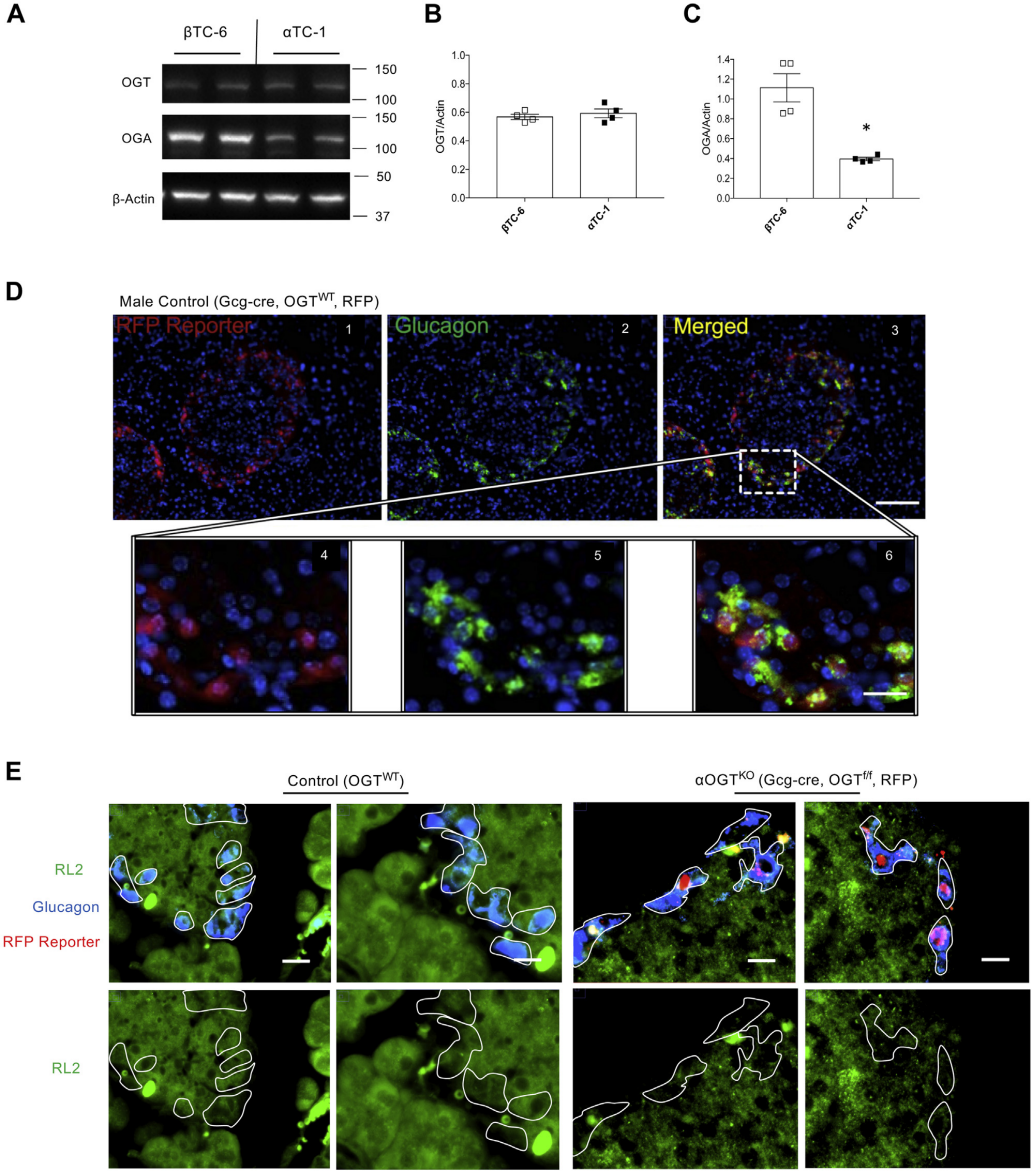
**OGT activity and presence of glucagon-*Cre* in α-cells.** Baseline levels of OGT and OGA protein (*A*) in αTC-1 and βTC-6 cells, assessed by western blot, and quantified and normalized to β-Actin (*B* and *C*, n = 4). Immunofluorescent staining for glucagon (*green*) and DAPI (*blue*) of fixed pancreatic sections at 10× magnification (scale = 100 μm) (*D* 1–3) and at 40× magnification (scale = 20 μm) (*D* 4–6), visualized in tandem with the endogenous tdTomato reporter (RFP). Immunofluorescent staining for glucagon (*blue*) and RL2 (*green*) (*E*), visualized in tandem with the endogenous tdTomato reporter (RFP) (scale = 10 μm) between control and αOGT^KO^ pancreas sections. Data represent mean ± SEM, n = 4 per group, ∗*p* < 0.05 compared with control. Analysis was done by unpaired, two-tailed Student’s *t*-tests.

High expression of OGT mRNA has been reported in the pancreas (12). Within the islet, it is controversial whether glucagon-producing α-cells or insulin-producing β-cells express more OGT mRNA (12, 15). Therefore, we first sought to compare protein levels of OGT and OGA between α-cells and β-cells cell lines due to the limited number and difficulty of selecting α-cells in primary islets. Baseline levels of OGT and OGA protein were measured in αTC-1 and βTC-6 immortalized cell lines (Fig. 1*A*). OGT levels were similar in both cell types in cell lines (Fig. 1*B*, Fig. S1, *A* and *B*). However, there was a significantly lower level of OGA protein present in the αTC-1 cell line compared with the βTC-6 cell line (Fig. 1*C*, Fig. S1, *A* and *C*). Interestingly, the level of O-GlcNAcylation, measured by the RL2 antibody, was comparable between both cell lines (Fig. S1*D*). RL2 is a specific O-GlcNAc antibody that has been validated in our lab (18) and others (19, 20). Together, these data show that OGT and OGA are expressed in α-cells, and thus the role of O-GlcNAcylation in α-cell physiology should be tested *in vivo*.

Therefore, we next tested the hypothesis that OGT is required for the maintenance of α-cell mass and for appropriate α-cell function *in vivo*. We utilized genetic manipulation to generate a mouse model with α-cell specific OGT deletion *in vivo*. αOGT^KO^ mice were produced by crossing Gcg-Cre mice (constitutive KO) with OGT^flox/flox^ mice. To facilitate lineage tracing, αOGT^KO^ mice were also crossed with a fluorescent *Cre* reporter (either RFP or GFP) to mark all cells exhibiting the *Cre* activity. The Gcg-*Cre* recombination efficiency was previously calculated at 94 to 97% of α-cells, whereas it was detected in a negligible (∼0.2%) proportion of β-cells (21). In our hands, we detected RFP, by immunofluorescent staining, colocalized with glucagon-expressing cells of the islet in Gcg-cre, OGT^WT^ mice (Fig. 1*D*). We then compared the staining patterns in Gcg-cre, OGT^WT^ mice with those in αOGT^KO^ mice. In addition, we conducted RL2 staining to test whether genetic deletion of OGT in the α-cells had an effect on O-GlcNAcylation. At 1 month of age, αOGT^KO^ mice showed reduced RL2 staining in glucagon-positive cells in comparison with the control (Fig. 1*E*). Importantly, the glucagon-positive cells with reduced RL2 staining were also positive for RFP, suggesting activation of the Gcg-*Cre* in these cells. These data confirmed that our αOGT deletion resulted in a decrease in α-cell O-GlcNAcylation.

### Reduced nonfasted serum glucagon levels in αOGT^KO^ mice do not affect glucose homeostasis

**Figure 2:**
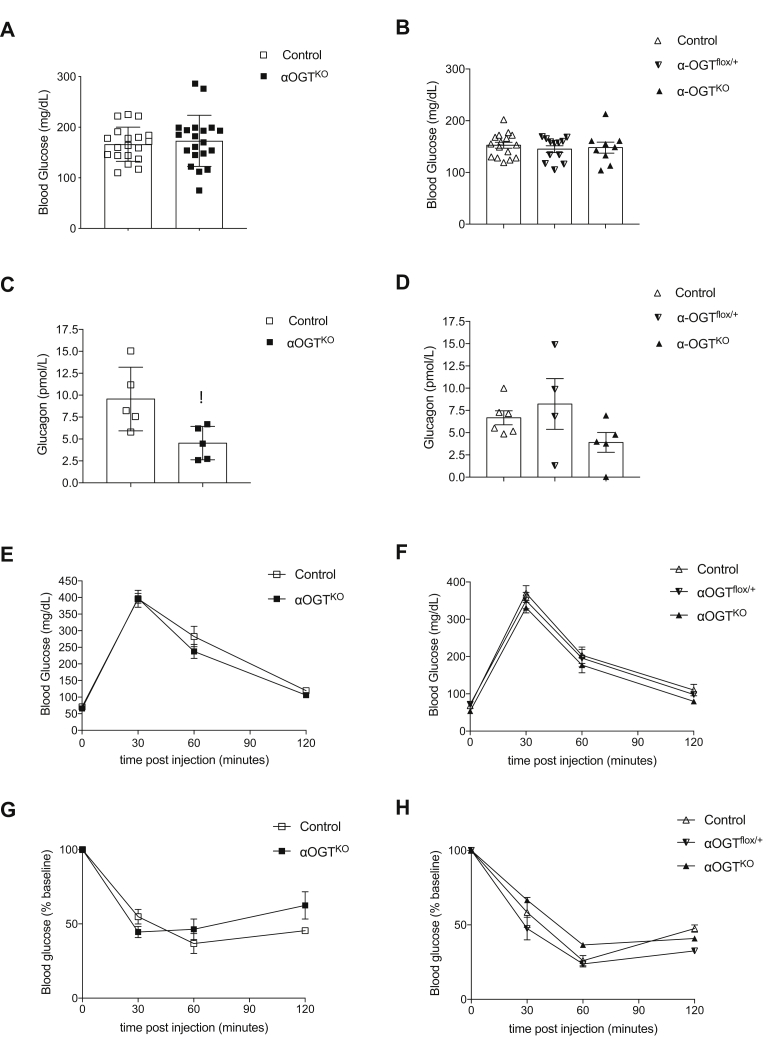
**αOGT**^**KO**^**mice exhibit normal glucose homeostasis and β-cell function, despite reduced random circulating glucagon levels in fed conditions.** Random fed blood glucose levels in male mice (*A*), (n = 20 per group) and female mice (*B*), (n = 9–15 per group (control, partial αOGT^flox/+^, or full αOGT^KO^)). Random fed circulating glucagon levels in male (*C*), (n = 5 per group) and female mice (*D*), (n = 4–6 per group). Intraperitoneal glucose tolerance test in 3-month-old male (*E*), (n = 7 per group) and female mice (*F*), (n = 8 per group). Glucose levels after intraperitoneal injection of insulin in 3-month-old male (*G*), (n = 6 per group) and female mice (*H*), (n = 5 per group). Data represent mean ± SEM, ∗*p* < 0.05 compared with control. Analysis was done by unpaired, two-tailed Student’s *t*-tests and two-way ANOVA.

After confirming that OGT deletion reduced O-GlcNAcylation in α-cells, we next sought to assess the metabolic health of the αOGT^KO^ mice in fed and fasted states, in order to determine what effect this deficit has on islet function. In nonfasted states, male and female αOGT^KO^ mice showed normal blood glucose levels (Fig. 2, *A* and *B*) as well as no alterations in serum insulin levels, compared with controls (Fig. S2, *A* and *B*). When we analyzed serum for glucagon levels, however, male αOGT^KO^ mice displayed significantly reduced levels of serum glucagon in the fed state (Fig. 2*C*). Comparable level of glucagon was observed in female αOGT^HET^ and control (Fig. 2*D*). Female αOGT^KO^ mice showed a trend toward reduction of serum glucagon levels in nonfasted state, but the results did not reach significance (Fig. 2*D*). Both male and female αOGT^KO^ mice displayed normal glucose tolerance (Fig. 2, *E* and *F*). Additionally, neither group showed differences in insulin sensitivity *via* IP insulin tolerance test (Fig. 2, *G* and *H*). These results show that in spite of their reduced serum glucagon levels, αOGT^KO^ mice manage to maintain normal glucose homeostasis.

### Reduced circulating glucagon response to fasting challenge and altered pyruvate tolerance in αOGT^KO^ mice

**Figure 3:**
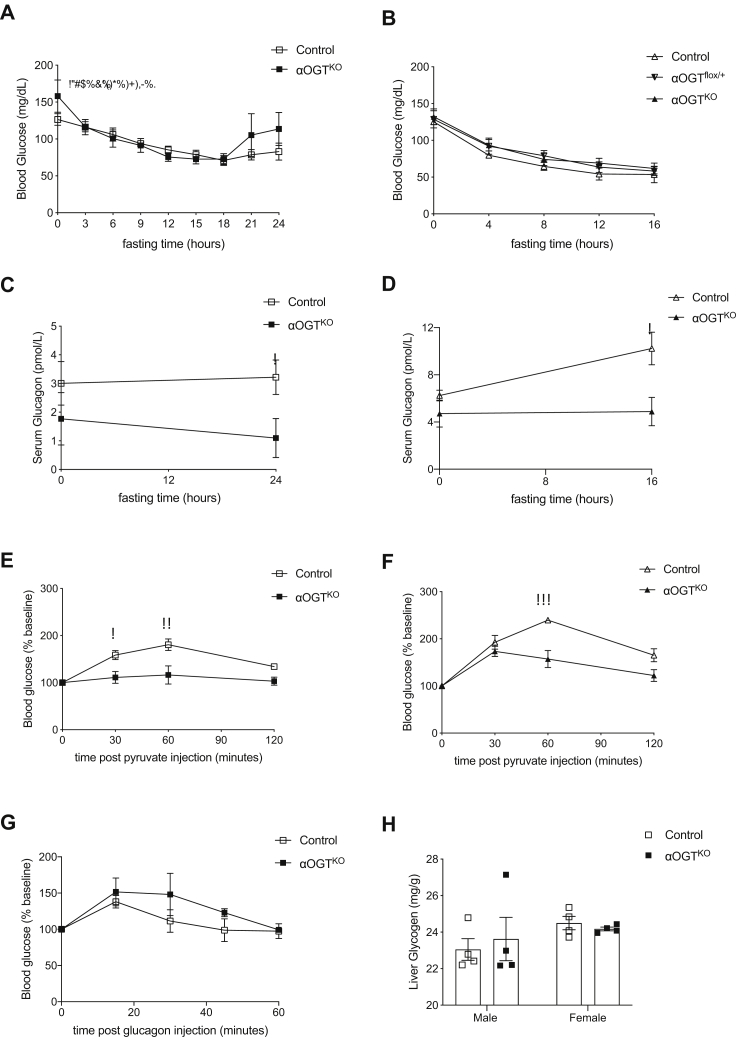
**αOGT**^**KO**^**mice exhibit reduced fed and fasting glucagon levels and decreased hepatic glucose output but remain euglycemic under fed and fasted conditions**. Fasting experiment conducted in 4-month-old male mice (*A*) for 24 h (n = 10 per group) and in 4-month-old female mice (*B*) for 16 h (n = 6 per group). Circulating plasma glucagon levels in 4-month-old male mice in duration of the fasting experiment from “A” at timepoint 0 and 24 (*C*) and in 4-month-old female mice in duration of the fasting experiment from “B” at timepoint 0 and 16 (*D*). Glucose levels after intraperitoneal injection of pyruvate in 3-month-old males (*E*), (n = 6 per group) and in 3-month-old females (*F*), (n = 6 per group). Glucose levels after intraperitoneal injection of glucagon in 3.5-month-old males (*G*), (n = 6 per group). Liver glycogen assessment in 6-month-old male and female mice (*H*), (n = 4 per group). Data represent mean ± SEM, ∗*p* < 0.05, ∗∗*p* < 0.01, ∗∗∗*p* < 0.001 compared with control. Analysis was done by unpaired, two-tailed Student’s *t*-tests, repeated measures one-way ANOVA, and two-way ANOVA.

Next, we challenged the αOGT^KO^ mice by performing a fasting experiment. Male control and αOGT^KO^ mice showed similar decrease in blood glucose after 18 h of fasting (Fig. 3*A*). Control and αOGT^KO^ blood glucose levels did level off and even begin to increase between 18 and 24 h of fasting, and there was a trend toward higher increase in the αOGT^KO^ (Fig. 3*A*). Female control and partial or full αOGT^KO^ mice also showed similar decrease in blood glucose after 16 h of fasting, with no marked differences in glucose level (Fig. 3*B*). During this fasting period, male αOGT^KO^ circulating glucagon levels were significantly lower than the control (Fig. 3*C*). Female αOGT^KO^ circulating glucagon levels were significantly lower at the 16-h fasted timepoint (Fig. 3*D*). These knockout mice failed to increased serum glucagon levels in response to the fasting. When subjected to an IP pyruvate tolerance test, which measures the gluconeogenesis capacity of the mice in a hypoglycemic condition, both male (Fig. 3*E*) and female (Fig. 3*F*) αOGT^KO^ mice exhibited significantly reduced blood glucose in response to pyruvate injection compared with the control, indicating impaired gluconeogenesis. Four-month-old male αOGT^KO^ mice exhibited normal glucagon sensitivity (Fig. 3*G*). Liver glycogen measurement in both male and female mice showed no significant differences (Fig. 3*H*). These results show that αOGT^KO^ mice were able to maintain normal glucose homeostasis, despite evidence of reduced glucagon levels in the circulation.

### αOGT^KO^ mice show reduced α-cell mass

**Figure 4:**
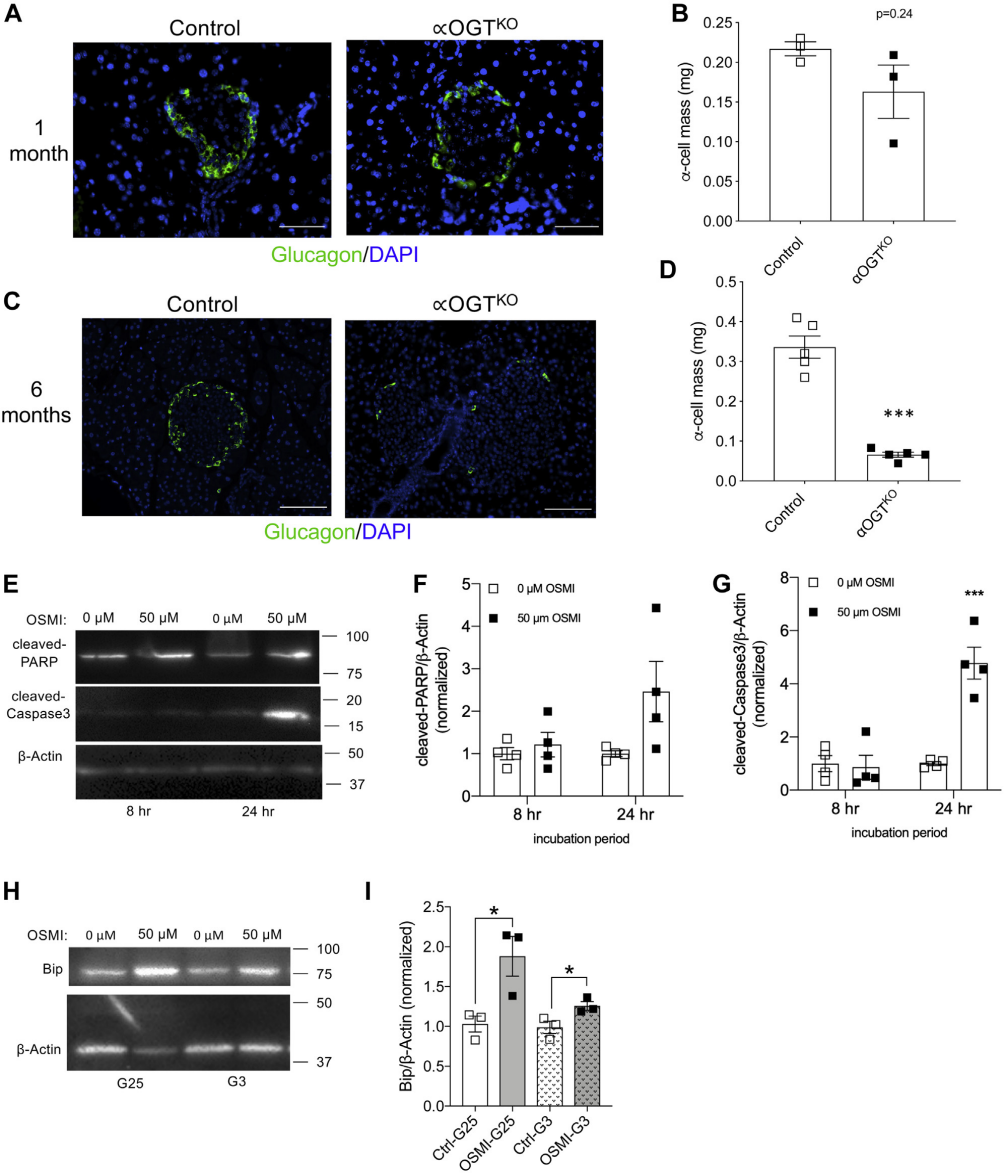
**αOGT**^**KO**^**mice show significant reduction in α-cell mass at 6 months**. Immunofluorescent staining for glucagon (*green*) and DAPI (*blue*) of fixed pancreatic sections at 10 to 20× magnification, in 1-month (*A*) and 6-month-old male mice (*C*), (scale = 50 μm). Quantification of α-cell mass at 1 month (*B*), (n = 3 per group) and 6 months of age in male mice (*D*), (n = 5 per group). Protein levels of cleaved-PARP and cleaved-Caspase-3 in αTC-1 cells treated with OSMI-1 inhibitor or DMSO (control) for 8 and 24 h, assessed by western blot (*E*), quantified and normalized to B-actin (*F* and *G*) (n = 4). Protein levels of Bip in αTC-1 cells treated with OSMI-1 inhibitor or DMSO (control) in 3 or 25 mM glucose for 24 h, assessed by western blot (*H*), quantified and normalized to B-actin (*I*) (n = 3). All scale bars = 50 μm. Data represent mean ± SEM, ∗*p* < 0.05, ∗∗*p* < 0.01, ∗∗∗*p* < 0.001 compared with control. Analysis was done by unpaired, two-tailed Student’s *t*-tests.

After observing reduced glucagon levels in αOGT^KO^ mice, we determined whether loss of α-cell mass can contribute to this effect. Morphometric analysis of 1-month-old male αOGT^KO^ pancreata demonstrated that α-cell mass at postweaning age was not statistically different between genotypes (Fig. 4, *A* and *B*). However, α-cell mass was significantly decreased in 6-month-old male αOGT^KO^ mice with an average mass of approximately 0.08 mg in the αOGT^KO^, compared with an average of approximately 0.33 in the control (Fig. 4, *C* and *D*). Representative images of the whole pancreas were shown in Fig. S3, *A* and *B*. Based on these data, we hypothesized that α-cells were diminishing over time in αOGT^KO^ mice. Due to the heterogeneity of islet cells, and limitations imposed by using primary islets from αOGT^KO^ mice (reduced number of α-cells in αOGT^KO^ islets), we used αTC-1 cells to assess the effect of OGT inhibition on α-cell death. We used the OGT inhibitor, OSMI-1 (50 μM), to reduce O-GlcNAcylation in αTC-1 cells. OSMI-1 treatment of αTC-1 cells decreased O-GlcNAcylation measured at both 8 and 24 h timepoints compared with control (DMSO) samples (Fig. S4*A*), demonstrating successful reduction of O-GlcNAcylation. We then assessed the protein level of apoptosis markers, cleaved-caspase-3 and cleaved-Poly (ADP-ribose) polymerase (PARP) (Fig. 4, *E*–*G*). During apoptosis, PARP, a nuclear DNA-binding protein, which detects DNA strand breaks and performs in base excision repair, is cleaved by caspase-3. Here, we observed a trend of increased levels of cleaved-PARP and a significant increase in cleaved-caspase-3 level after 24 h treatment with OSMI-1. We previously reported that β-cell OGT-deficient cells display ER stress-mediated apoptosis (14), and we show in OGT inhibited αTC-1 cells (Fig. S4*B*), induction of Bip, a marker of ER stress (Fig. 4, *H* and *I*). These data suggest that increased ER stress may contribute to cell death in αTC-1 cells when O-GlcNAcylation is blocked, consistent with the reported effects of OGT inhibition in other pancreatic cells (14, 16) *in vivo*.

### αOGT^KO^ mice show reduced single-cell glucagon content and impaired islet glucagon secretion

**Figure 5:**
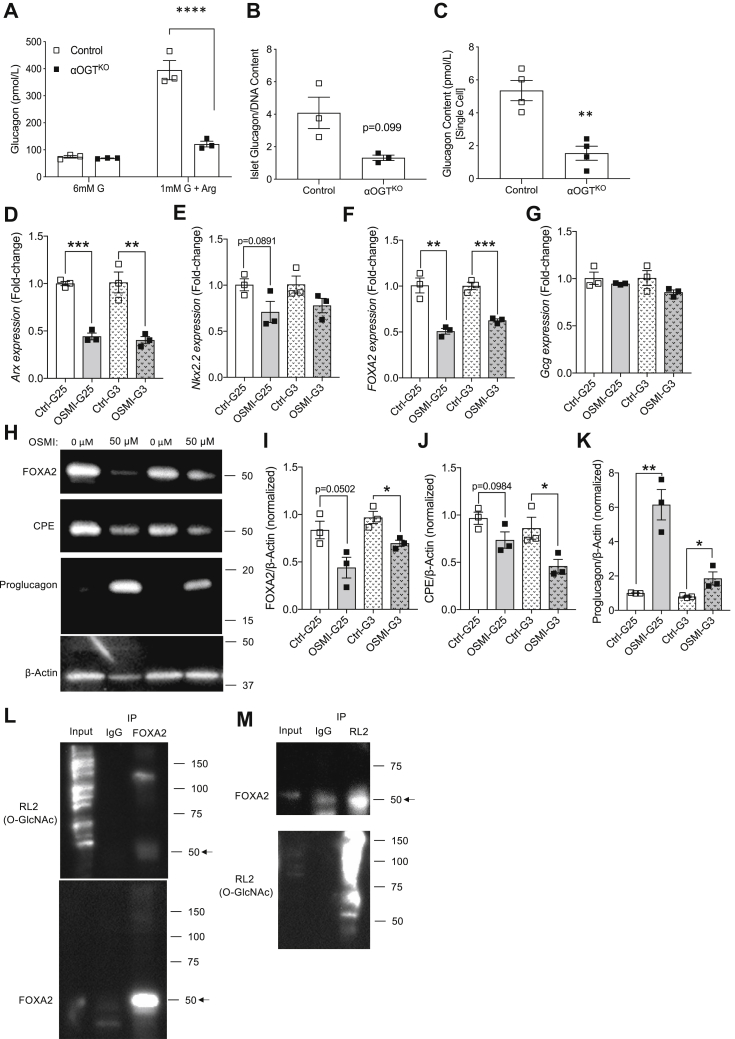
**αOGT**^**KO**^**mice show significant deficiency in islet glucagon content, single cell glucagon content, and *in vitro* glucagon secretion.***In vitro* glucose inhibited glucagon secretion in male mice (*A*), (n = 3 per group), islet glucagon content normalized by islet DNA content (*B*), and single α-cell glucagon content (*C*), (n = 4 per group). qPCR assessments of Arx (*D*), Nkx2.2 (*E*), FOXA2 (*F*), and Gcg (*G*) transcripts and western blot assessments (*H*, note that 4H and 5H are from the same western blot membrane, and thus, 4H actin is reused here) of FOXA2 (*I*), CPE (*J*), proglucagon (*K*) proteins from OSMI-treated αTC-1 cells treated with OSMI-1 inhibitor or DMSO (control) in 3 or 25 mM glucose for 24 h (n = 3). Immunoprecipitation of FOXA2, followed by immunoblot against O-GlcNAc (RL2) (*L*) and immunoprecipitation of O-GlcNAcylated proteins (*via* RL2 antibody), followed by immunoblot against FOXA2 (*M*) (n = 2 each).

After establishing the effect of OGT loss on α-cell mass, we next sought to determine the effect on α-cell function. To assess α-cell function, we subjected islets from 2-month-old male mice to an *in vitro* glucose and arginine-inhibited glucagon secretion test *in vitro*. Younger mice were used for this test in order to avoid major confounding effect of reduced α-cell number present per islet, since our data show a severe reduction of α-cell mass in 6-month-old mice. Islets isolated from 2-month-old male αOGT^KO^ mice showed a significant impairment in glucagon secretion compared with control, as shown by the significantly lower amount of glucagon released when islets were treated with 1 mM glucose +20 mM arginine condition for 1 h (Fig. 5*A*). A trend toward a lower level of total islet glucagon content (normalized by islet DNA) was also observed (Fig. 5*B*). This indicated to us that the secretory defect in αOGT^KO^ islets was not likely due to a defect in secretory mechanism, but rather could be attributed to lower glucagon content per single α-cell in the αOGT^KO^ islets. Assessment of total pancreas glucagon content was not ideal due to the defect in α-cell mass. Therefore, to directly assess glucagon content at the single cell level, we dispersed islets from αOGT^KO^ mice with GFP reporter for visualization and hand-picked GFP cells under the microscope (Fig. S5*A*). We determined that the averaged glucagon content per cell in αOGT^KO^ mice was significantly reduced compared with that of the control cells (Fig. 5*C*). To test whether glucagon biosynthesis is altered in the islets, we assessed for glucagon transcript and observed a reduction in i-αOGT^KO^ islets (Fig. S4*C*). We also treated αTC-1 cells acutely with OSMI-1 as before. We found decreased mRNA levels of α-cells transcription factors (Arx, Nkx2.2, and FOXA2, Fig. 5, *D*–*F*) as well as protein level of FOXA2 in OGT-inhibited αTC-1 cells (Fig. 5, *H* and *I*). However, no significant reduction in glucagon mRNA level was detected in the OSMI-1-treated αTC-1 cells (Fig. 5*G*). Then, we assessed the protein level of the prohormone convertase, Carboxypeptidase E (CPE), which was shown to regulate total glucagon content in αTC-1 cells (22), and found it decreased in OSMI-1 treated αTC-1 cells (Fig. 5, *H* and *J*). Decreased CPE protein was correlated with the elevated proglucagon levels by western blot (Fig. 5, *H* and *K*), which suggested a defect in glucagon processing. Although glucagon transcription was not altered in acute treatment of αTC-1 cells with OSMI-1, glucagon mRNA was reduced in islets of iαOGT^KO^ mice. Next, we aimed to test whether FOXA2 was O-GlcNAc-modified in α-cells. FOXA2, a potent regulator of glucagon transcription, was projected to be O-GlcNAc by a prediction software (23), at multiple potential O-GlcNAc sites. In αTC-1 cells, we confirmed that FOXA2 was indeed an OGT target, *via* IP pull-down of the protein (Fig. 5, *L* and *M*). Altogether, these findings illustrate the resultant deficiencies of αOGT^KO^, consisting of reduced α-cell mass in older mice and a deficiency in islet glucagon secretion and single α-cell content in younger mice.

### I-αOGT^KO^ mice display reduced circulating glucagon and impaired pyruvate tolerance, with no effect on blood glucose level

**Figure 6:**
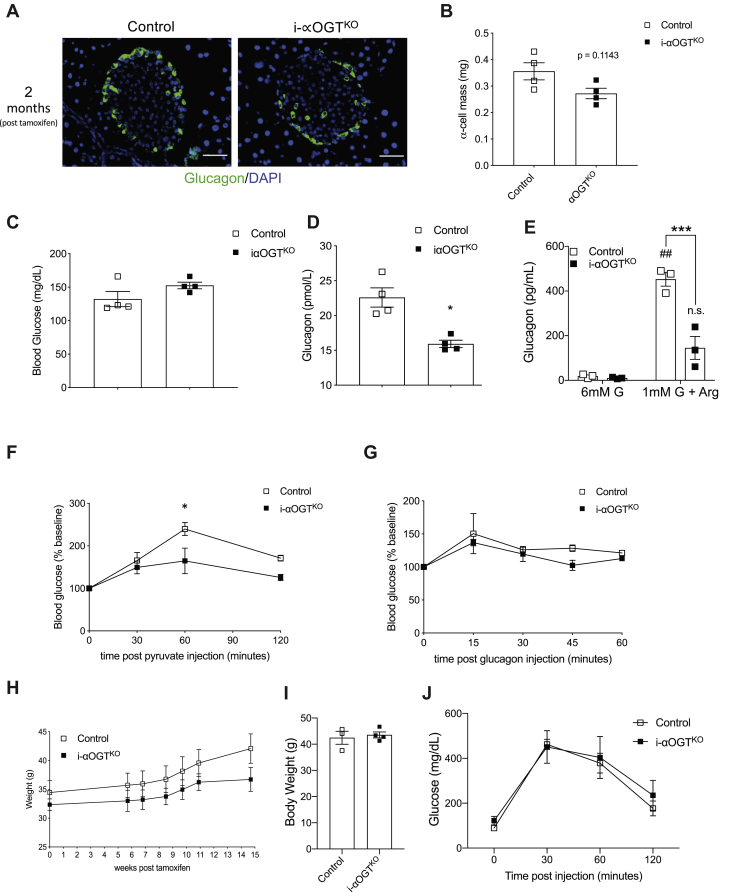
**i-αOGT**^**KO**^**mice show trend of reduced α-cell mass, as well as reduced circulating glucagon and impaired PTT.** Immunofluorescent staining for glucagon (*green*) and DAPI (*blue*) of fixed pancreatic sections at 20× magnification, in male control and i-αOGT^KO^ mice at 15 weeks post-tamoxifen treatment (scale = 50 μm) (*A*). Quantification of α-cell mass in male control and i-αOGT^KO^ mice 15 weeks post-tamoxifen treatment (*B*), (n = 4 per group). Random fed blood glucose levels in male control and i-αOGT^KO^ mice 2 months post-tamoxifen treatment (*C*), (n = 4 per group). Random fed circulating glucagon levels in male control and i-αOGT^KO^ mice 2 months post-tamoxifen treatment (*D*), (n = 4 per group). *In vitro* glucose inhibited glucagon secretion in male mice (*E*), (n = 3 per group). Glucose levels after intraperitoneal injection of pyruvate in male control and i-αOGT^KO^ mice 2 months post-tamoxifen treatment (*F*) (n = 4 per group). Intraperitoneal glucagon challenge in male control and i-αOGT^KO^ mice 2 months post-tamoxifen treatment (*G*) (n = 4 per group). Body weight measured in male i-αOGT^KO^ mice (*H*) (n = 4 per group) from 0 to 15 weeks or in a different cohort of mice at 24 weeks (*I*, n = 4) post-tamoxifen treatment. IPGTT done at 24 weeks (*J*, n = 4) post-tamoxifen treatment. Data represent mean ± SEM, ∗*p* < 0.05, ∗∗*p* < 0.01, ∗∗∗*p* < 0.001 compared with control. Analysis was done by unpaired, two-tailed Student’s *t*-tests, repeated measures one-way ANOVA, and two-way ANOVA.

αOGT^KO^ mice displayed reduced α-cell mass, reduced glucagon levels, and impaired gluconeogenesis. This, however, did not affect their ability to maintain normal blood glucose levels. Next, we sought to corroborate these findings in the inducible i-αOGT^KO^ model, thus observing the effect of acute OGT deletion in adult mice. We generated i-αOGT^KO^ animals by crossing male Gcg-Cr*e*^ERTM^ (21) (inducible KO) and female with OGT^flox/flox^ mice. Morphometric analysis of i-αOGT^KO^ mouse pancreata was conducted at 15 weeks post-tamoxifen induction (Fig. 6*A*). Expression of RFP cells in glucagon cells demonstrated the efficiency of the tamoxifen in inducing Cre recombination (Fig. S5*B*). We assessed α-cell mass at 15 weeks post-tamoxifen, and we uncovered that i-αOGT^KO^ and age-matched control mice show comparable α-cell mass (Fig. 6*B*). I-αOGT^KO^ mice demonstrated normal blood glucose level (Fig. 6*C*), fasting and fed serum insulin (Fig. S6, *A* and *B*) compared with the control. In line with the constitutive αOGT^KO^ model, i-αOGT^KO^ also displayed a significantly reduced random blood glucagon levels (Fig. 6*D*). The defect in glucagon secretion from islets of i-αOGT^KO^ *in vitro* was confirmed (Fig. 6*E*), as well as loss of glucagon content (Fig. S6*C*). Additionally, iαOGT^KO^ mice exhibited significant pyruvate intolerance (Fig. 6*F*) and normal glucagon sensitivity (Fig. 6*G*). No body weight phenotype was observed in the iαOGT^KO^. Body weights of experimental mice (two different cohorts) at 15 or 24 weeks post-tamoxifen induction showed no significantly different increase in body weight than the age-matched controls (Fig. 6, *H* and *I*, Fig. S6, *D* and *E*). Glucose tolerance was also noted to be comparable between the control and iαOGT^KO^ at 24 weeks post-tamoxifen induction (Fig. 6*J*, Fig. S6*F*). These findings demonstrated that acute deletion of OGT in adult mice had a similar effect on glucagon level but differed from αOGT^KO^ through its milder effect on α-cell mass.

### αOGT^KO^ male, but not female, mice exhibit obesity due to hyperphagia

**Figure 7:**
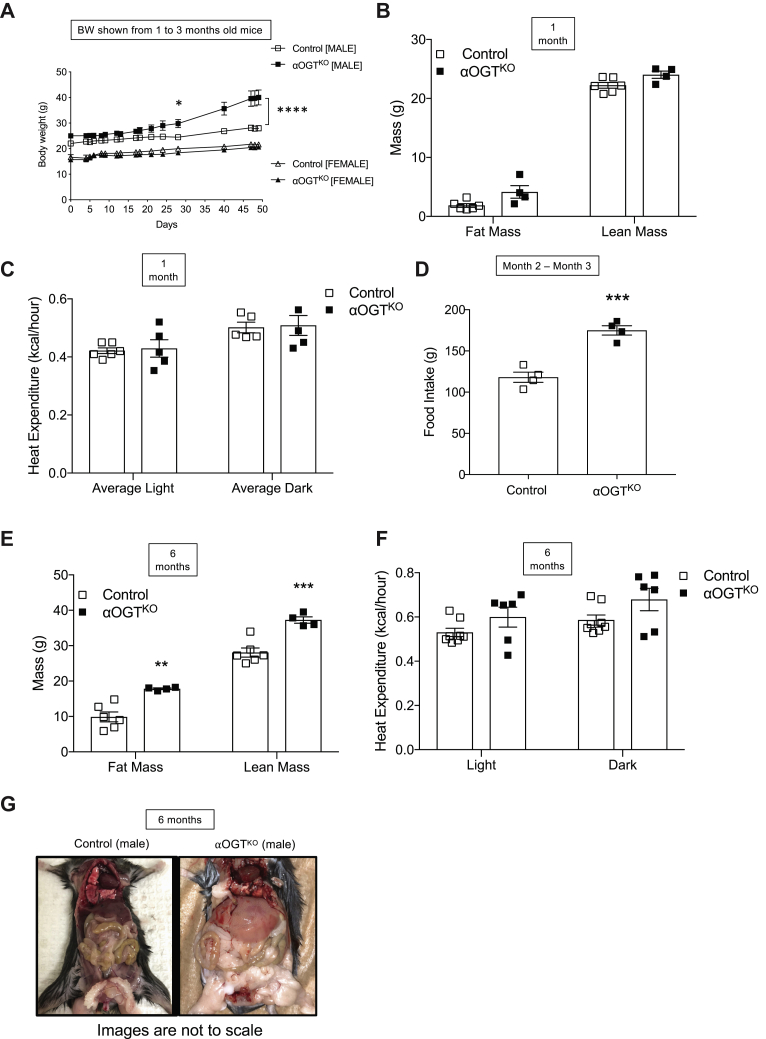
**Male αOGT**^**KO**^**mice exhibit hyperphagic-induced obesity, but females and male i-αOGT**^**KO**^**mice are protected.** Body weight in male and female mice between 1 and 3 months of age (*A*), (n = 5 per group). MRI body scan of male mice at 1 month of age (*B*, n = 4–5 per group). Heat expenditure assessment of male mice at 1 month of age (*C*) (n = 5 per group). Food intake in male mice (n = 4) from 2 to 3 months of age (*D*), assessed in total grams of food consumed that month. MRI body scan of male mice at 6 months of age (*E*, n = 6 per group) and heat expenditure (*F*, n = 6) calculated using ANCOVA analysis of covariate means to account for difference in body weight. Fatty deposit visualization in male 6-month-old control and αOGT^KO^ mice; images are not to scale (*G*). Data represent mean ± SEM, ∗*p* < 0.05, ∗∗*p* < 0.01, ∗∗∗*p* < 0.001 compared with control. Analysis was done by unpaired, two-tailed Student’s *t*-tests, repeated measures one-way ANOVA, and two-way ANOVA.

Unlike the iαOGT^KO^ model, the αOGT^KO^ mice displayed a body weight phenotype. Weekly weighing of mice under normal chow diet revealed heavier body weight over time in male αOGT^KO^ mice, which became significant at 2 months of age and continued to increase in until 3 months of age, body weight measurement from 1 month to 3-month-old mice is shown (Fig. 7*A*). In sharp contrast, αOGT^KO^ and control females did not show differences in body weight (Fig. 7*A*). To analyze contributing body mass differences in these mice, we conducted an Echo-MRI imaging in young and old cohort of mice. MRI imaging of 1-month-old male αOGT^KO^ mice showed no significant differences in lean mass or fat mass (Fig. 7*B*). In addition, metabolic cage analysis in these mice showed no significant differences in heat expenditure between αOGT^KO^ and control mice (Fig. 7*C*). Food intake studies between 2 and 3 months of age revealed that αOGT^KO^ mice, at this timepoint, were eating a significantly higher average amount of chow per month (Fig. 7*D*). We also performed the metabolic cages study at 6 months of age, where male αOGT^KO^ mice had become significantly heavier than their control counterparts, with Echo-MRI analysis revealing significantly increased levels of both fat and lean mass (Fig. 7*E*). When corrected with body weight, there was a nonsignificant difference in heat expenditure between αOGT^KO^ and control mice (Fig. 7*F*). Consistent with the progression of obesity, we found normal circulating leptin level in 2 to 3 months old mice, but significantly increased leptin levels in 6-month-old αOGT^KO^ mice (Fig. S6, *G* and *H*). These findings show a phenotype of obesity in part due to hyperphagia and not due to defect in energy balance, and this was discovered only in male, but not in female αOGT^KO^ or in male i-αOGT^KO^ mice 3 months (Fig. 6*H*) or 5 months post-tamoxifen injection (Fig. 6*I*). Visual differences in fat deposition between male αOGT^KO^ and control mice was observed in 6-month-old mice at the time of sacrifice (Fig. 7*G*).

### Male αOGT^KO^ mice show significant reduction in α-CaMKII-expressing cells in the PVN of obese αOGT^KO^ mice

**Figure 8:**
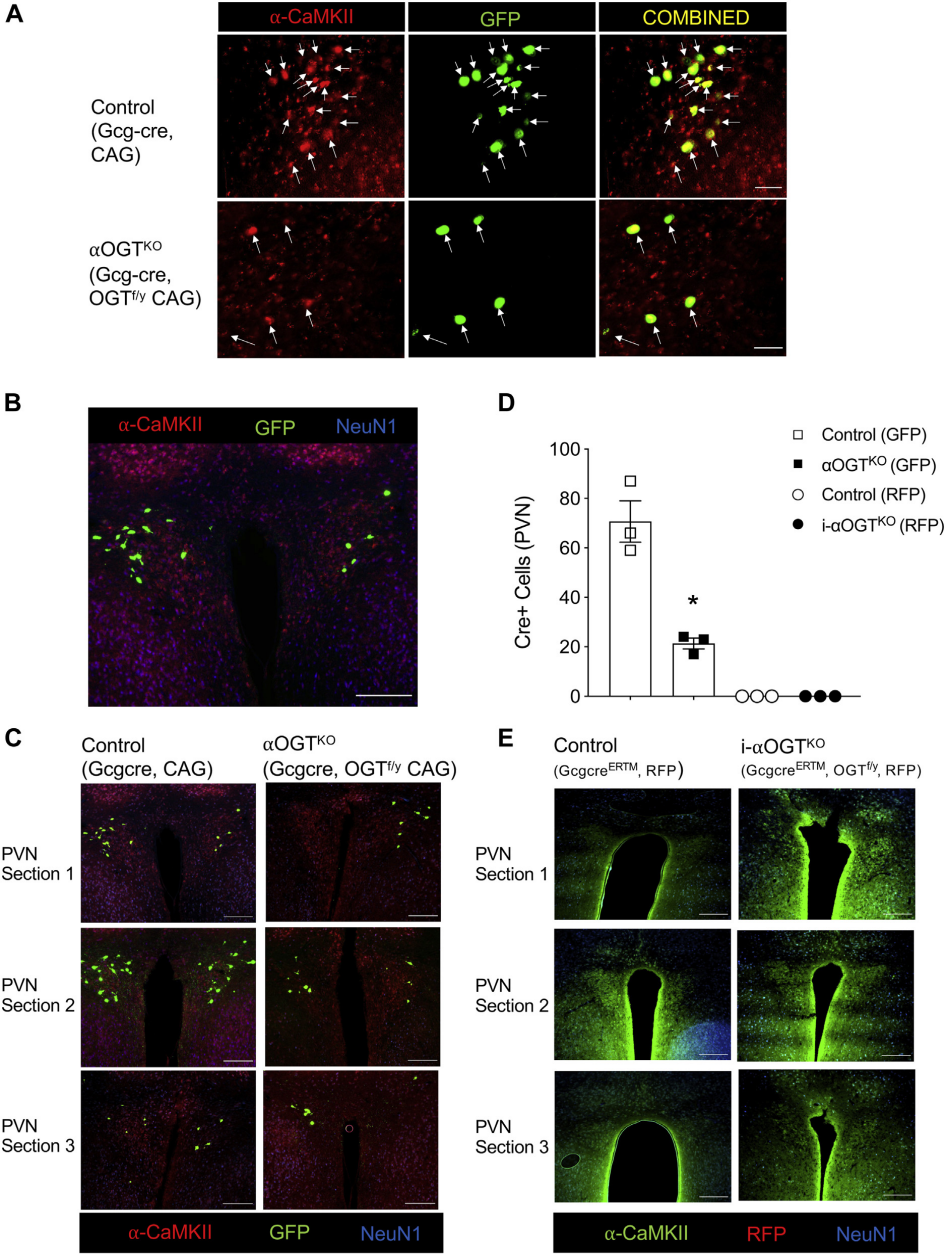
**Male αOGT**^**KO**^**mice show reduction in GFP-positive α-CaMKII-positive cells in the hypothalamic PVN.** Immunofluorescent staining for α-CaMKII (*red)*, in tandem with endogenous GFP *Cre* reporter (*green*) in 4-month-old male mouse fixed coronal brain sections (*A*). (scale = 50 μm). 10× image of PVN in a 4-month-old Gcg-cre, OGT^WT^; CAG-GFP mouse, stained for α-CaMKII (*red*) and NeuN1 (*blue*), in tandem with endogenous GFP (*green*) (*B*). (scale = 200 μm). Immunofluorescent PVN staining of male control and αOGT^KO^ mice for α-CaMKII (*red*) and NeuN1 (*blue*), in tandem with endogenous GFP (*green*) (*C*, *B* is reshown in *C* for a comparison as control). (scale = 200 μm). Full PVN *Cre*(+) cell number, per mouse brain, was visualized in three adjacent 40 μm sections. Quantification of *Cre*-positive cell number in PVN, totaled between three concomitant sections per mouse brain (*D*) (n= 3 per group). *Cre*-positive cells were only counted if colocalized with both α-CaMKII and NeuN1. Immunofluorescent PVN staining of male control and i-αOGT^KO^ mice for α-CaMKII (*green*) and NeuN1 (*blue*), in tandem with endogenous RFP (*red*) (*E*). (scale = 200 μm). Data represent mean ± SEM, ∗*p* < 0.05, ∗∗*p* < 0.01, ∗∗∗*p* < 0.001 compared with control. Analysis was done by unpaired, two-tailed Student’s *t*-tests.

Next, we sought to determine the mechanisms behind the obesity in αOGT^KO^ mice. Deletion of OGT in α-CaMKII-positive periventricular nucleus (PVN) neurons in the hypothalamus has been previously shown to cause obesity in part due to hyperphagia (24). After observing hyperphagia-induced obesity in the αOGT^KO^ mice, we hypothesized that deletion of OGT was occurring in the PVN. Indeed, immunofluorescent imaging of the hypothalamus of control mice expressing Gcg-*Cre* GFP-reporter showed the presence of GFP in the PVN (Fig. 8, *A* and *B*). GFP-positive cells in the PVN showed consistent colocalization with α-CaMKII, suggesting that *Cre*-mediated OGT deletion was occurring in α-CaMKII-positive neurons in the hypothalamus (Fig. 8, *A* and *B*). Comparison of Gcg-*cre* expression (assessed by GFP or RFP-positive expression in neurons) in the PVN revealed significantly reduced number of Gcg-*Cre* positive cells in male αOGT^KO^ mice compared with controls (Fig. 8, *C* and *D*), suggesting a possible defect on survival of the target cells, in line with previous report in neurons (25) and pancreatic cells (14, 16). No evidence of Gcg-*Cre*^ERTM^ activity, indicated by RFP-reporter, was observed in the PVN of i-αOGT^KO^ mice or corresponding OGT^WT^ controls (Fig. 8, *D* and *E*), suggesting that OGT levels may have not been altered and thus the lack of effect in body weight in these mice (Fig. 6, *H* and *I*). However, RFP was only detected in the α-cells in the pancreas of these mice (Fig. S5*B*). It is important to point out that *Gcg*^*CreERT2*^ knockin mice is more pancreas-specific and brain expression is limited to the NTS region (21). In sum, these findings show a Gcg-*Cre*-mediated reporter activation in the brain and thus suggest an OGT deletion in the PVN of the constitutive αOGT^KO^ mice. Moreover, we demonstrated that OGT deletion resulted in loss of GFP(+)/α-CaMKII(+) cell number in these mice compared with control.

## Discussion

As a nutrient sensor, OGT is highly expressed in the pancreas and uniquely poised to orchestrate cell signaling pathways that impact growth and survival, in response to nutrient availability (26). The pancreatic islets sense changes in nutrients during the cycles of feeding and fasting, and they release insulin and glucagon respectively to maintain glucose homeostasis. Our group has recently shown that OGT plays an important role in β-cell health, and ablation of OGT in β-cells leads to diabetes and cell failure (17, 18). The role of OGT in α-cells has remained unknown. In the present study, we determined that OGT plays an important role in the regulation of α-cell mass and function. Loss of OGT resulted in loss of α-cell mass and dysfunctional glucagon secretion in mice.

The high expression of OGT in α-cells has been previously reported (12), and this is supported by our data showing that OGT protein was expressed at comparable level between αTC-1 and βTC-6 cells. Interestingly, a significantly lower expression of OGA protein was found in αTC-1 cells compared with βTC-6 cells. OGA’s mechanistic action is opposite to that of OGT, removing UDP-GlcNAc substrates from previously O-GlcNAcylated proteins (15). Lower level of counterregulatory OGA protein in αTC-1 cells compared with βTC-6 cells, despite similar OGT level, may suggest a higher O-GlcNAcylation of proteins in α-cells. However, we observed comparable level of O-GlcNAcylaiton in α-cells and βTC-6 cells. Thus, future study can further investigate and compare OGT target proteins and their patterns of O-GlcNAcylation in both endocrine cells, which may provide insights of the O-GlcNAcylation status in α-cells *versus* β-cells.

The biological impact of O-GlcNAcylation on α-cell mass and function has not been investigated *in vivo*. In the current paper, we describe mild but important phenotypes in animals lacking OGT in their α-cells constitutively (αOGT^KO^) or acutely (i-αOGT^KO^). Through immunofluorescent staining *in vivo*, we demonstrated the efficiency of the Cre in the αOGT^KO^ and i-αOGT^KO^ through colocalization of the RFP or GFP reporter with glucagon within the islets. Shiota *et al.* (21) have also reported RFP expression in ∼95% of α-cells and negligible (∼0.2%) expression in β-cells. The normal glucose tolerance and insulin sensitivity phenotype of young and old αOGT^KO^ mice support our finding that OGT was specifically deleted in α-cells, while leaving β-cells phenotypically normal. Deletion of OGT in β-cells causes glucose intolerance and overt diabetes at 6 weeks of age due to a significant loss of β-cell mass and insulin secretion dysfunction *in vivo* (14, 16, 18, 27). A major phenotype displayed by the αOGT^KO^ mice *in vivo* was the reduced fed-state circulating glucagon levels in 3-month-old mice. This deficiency in serum glucagon level was in part due to a significant reduction in glucagon secretion and islet glucagon content in the αOGT^KO^ mice. In older mice (6 months old), a significant reduction in α-cell mass in male αOGT^KO^ was observed, which is consistent with previous findings that OGT plays an essential role in the maintenance of endocrine islet cells’ survival (14, 16). The difficulty of working with pancreas *ex vivo* to assess proliferation and apoptosis in limited populations of α-cells has hampered this study. Blocking OGT action in β-cells and in pancreatic progenitors causes apoptosis (14, 16), and our present study also demonstrates that in α-cell lines, OGT regulates α-cells survival *via* apoptosis. The mechanisms behind reduction in glucagon content may in part be due to OGT’s regulation on key proteins important for α-cell health and function. For example, FOXA1 and FOXA2, both of which play an important role in α-cell glucagon biosynthesis and secretion (28), are O-GlcNAc modified by OGT in cancer cells, and this modification is important for the stability of the protein (29). Moreover, OGT has been shown to directly bind to the promoter of FOXA1 to regulate its expression levels (30). In the current study, we show that FOXA2 mRNA and protein levels were reduced in α-cell line with OGT inhibition. Moreover, we demonstrated that FOXA2 was O-GlcNAc modified in α-cells. However, it remains unclear if O-GlcNAcylation of FOXA2 in α-cells directly impacts its stability and/or function. The specific role of OGT in the expression and activity of other critical α-cell proteins is still relatively unknown. Future studies can be directed toward finding other OGT targets in α-cells.

Given our observations of reduced α-cell mass and function in older mice, we expected that αOGT^KO^ mice would encounter difficulty in maintenance of glucose homeostasis when challenged with hypoglycemic (fasted) conditions. Surprisingly, our data suggest that OGT activity in α-cells plays a minor to negligible physiological role in the regulation of glucose homeostasis. When subjected to fasting challenge, both male and female αOGT^KO^ mice showed normal glucose regulation despite significantly reduced circulating glucagon throughout the fasting experiment. These data suggest that partially preserved function of αOGT^KO^ cells is sufficient for glycemic control. In addition, αOGT^KO^ mice failed to increase blood glucagon levels in response to the increasingly hypoglycemic conditions. When subjected to pyruvate tolerance testing, αOGT^KO^ mice, as we expected, had significantly reduced glycemic response to pyruvate, indicating inability to promote gluconeogenesis. Additionally, no significant differences in glucagon sensitivity were observed in the αOGT^KO^ mice. These findings are similar to those made in reports such as Thorel *et. al*. and Bozadjieva *et. al.*, whose study showed that near-total α-cell ablation had no impact on blood glucose homeostasis (31, 32).

Constitutive genetic knockout of OGT in α-cells leads to a reduced α-cell mass that is age-dependent: reduced α-cell mass was observed in 6-month-old mice but not in 1-month-old animals. However, young and old islets from αOGT^KO^ mice displayed reduced α-cell function, including decreased serum glucagon levels, despite having no discernible effect on glucose homeostasis. Adult mice with an acute α-cell OGT deletion exhibited reduced levels of fed circulating glucagon while retaining normal α-cell mass. Similar to the constitutive model, i-αOGT^KO^ mice exhibited pyruvate intolerance, normal glucagon sensitivity, and decreased α-cell function. These findings mimic what has been observed between the β-OGT^KO^ and iβ-OGT^KO^ mice, where β-cell mass and insulin secretion defects were seen in β-OGT^KO^, but only functional defect in insulin secretion was observed iβ-OGT^KO^ (14).

The obesity phenotype in the constitutive male αOGT^KO^ mice fed standard chow diet is supported by the described effect of OGT in the PVN *in vivo*. Specific and acute deletion of OGT in α-CaMKII-positive neurons in the PVN of male animals has been shown to induce obesity through hyperphagia in mice (24). Morphometric analysis of α-CaMKII-positive with the GFP reporter suggested a significant reduction in the total numbers of these neurons in the male αOGT^KO^ mice, similar to a previous report (25). Thus, our finding is not surprising given that the glucagon gene is expressed in α-cells and other areas of the intestine and the brain (21, 33, 34, 35, 36). Cork *et al.* (37) reported expression of GLP-1 (indicative of preproglucagon transcription) in neurons of the NTS (brainstem), PVN, and VMH in the hypothalamus. We did not observe the obesity phenotype in female constitutive αOGT^KO^ mice, and we do not have an explanation why this occurred. The direct deletion of OGT in the PVN that is impacting satiety has only been studied in males and not females (24). Therefore, future studies can assess the mechanisms behind this sexual dimorphism phenotype. Using the inducible Cre to delete OGT, which has a higher fidelity to α-cell specifically (21), we did not observe any body weight phenotype. Both male and female i-αOGT^KO^ mice did not display obesity phenotype, and we observed undetectable level of the reporter RFP, a surrogate of *Cre* expression in the PVN, which suggested a more specific pancreatic Cre expression as previously demonstrated by Shiota *et al.* (21). Interpretation of the data should be cautioned because in the constitutive αOGT^KO^ mice, Cre-mediated OGT deletion may likely have occurred in all glucagon, GLP-1 and GLP-2 expressing cells. Relevant to the obesity phenotype, it is unlikely, however, that loss of OGT in the intestine contributed as deletion of OGT in intestine epithelial cells causes weight loss in mice (38). Ablation of OGT in AgRP neurons causes white adipose tissue browning and confers protection from diet-induced obesity (39). In the current study, we did not observe any alteration in heat expenditure in the constitutive αOGT^KO^ mice. Future studies will focus on identifying neurons that express OGT and related function.

In summary, these studies provide insights into the role of nutrient-driven pathways regulating glucagon secretion and α-cell mass. Our findings identify OGT as a critical nutrient sensor integrating signaling pathway necessary for glucagon secretion and in the regulation of α-cell mass maintenance.

## Experimental procedures

### Animals

The following mice were used in the current study: OGT^flox/flox^ mice (purchased from Jackson Laboratories), Gcg-Cre mice, harboring one allele of Cre recombinase driven by the glucagon promoter, and tamoxifen-inducible Gcg-CreERTM mice (gifts from George K. Gittes at the University of Pittsburgh, Pittsburgh, Pennsylvania, USA). We generated αOGT^KO^ (Gcg-cre; OGT^flox/y^ or OGT^flox/flox^), by crossing OGT^flox/flox^ females with and Gcg-Cre male mice and generated inducible-αOGT^KO^ (Gcg-creERTM; OGT^flox/y^ or OGT^flox/flox^) by crossing female OGT^flox/flox^ and male Gcg-creERTM mice. Reporter transgenic animals CAG-tdTomato (red fluorescent protein, RFP) and CAG–green fluorescent protein (GFP) were acquired from Dr Gittes and The Jackson Laboratory, respectively, and bred with αOGT^KO^ and i-αOGT^KO^ mice to endogenously report the presence of Cre recombinase activity. All mice were generated on a C57Bl/6J background and group housed on a 14:10 light–dark cycle. The multiple cohorts of mice were tested, but not all cohorts were tested or collected for the same experiments. For rigor, sex was considered as a variable, and sex of the mice was indicated in the figure legend and/or on the graph labels. Not all animals are tested for all experiments. Unless otherwise stated, controls used are littermates OGT^flox/flox^ mice. All studies were approved by the Institutional Animal Care and Use Committee (protocol #1806-36072A) at the University of Minnesota.

### Metabolic studies

Body weight and random blood glucose were monitored monthly for a total of 6 months. Blood was obtained from the tail vein, and blood glucose was measured with an Accu-Chek blood glucose meter. Serum glucagon levels were measured with ELISA. Intraperitoneal glucose tolerance test (IPGTT) (2 g/kg) and insulin tolerance test (ITT) (0.75 U/kg) were conducted as previously described (14). IP glucagon challenge (16 μg/kg) was performed by intraperitoneal injection of glucagon in 4- to 6-h fasted male mice. Fasting experiments were conducted by starting fast at 5 PM and monitoring blood glucose level every 2 h for either a 16 or 24 h period. Plasma glucagon levels were measured at timepoint 0, as well as the last timepoint (hour 16 or hour 24). Hepatic glucose production was measured by intraperitoneal injection of pyruvate (PTT) (2 g/kg) in 16- fasted male mice.

### Body weight analysis, calorimetry, and food intake

Echo-MRI (Echo Medical Systems LLC) and heat-expenditure analysis (Oxymax/CLAMS Lab Animal Monitoring System, Columbus Instruments) were conducted at the Integrative Biology and Physiology (IBP) Core at the University of Minnesota. Heat expenditure was measured in kcal/h over a 4-day period. Heat expenditure data in mice with significantly different body weights was adjusted by use of ANCOVA analysis done by the IBP Core. Food intake was determined by measuring daily amount of chow consumed by single-caged mice over a 1-month period. Circulating leptin levels were measured from serum collected from 2- to 3- and 6-month-old mice and assessed with Leptin ELISA (Alpco).

### Immunofluorescence, cell morphometry, and α-cell mass

Formalin-fixed and paraffin-embedded pancreata were sectioned (5 μM-thickness). Pancreas sections were prepared for staining with deparaffinization and blocking as previously described (16). Briefly, pancreatic sections were incubated overnight with primary antibody against glucagon, RL2, or insulin followed by secondary antibodies conjugated to fluorophores and DAPI solution. Stained slides were imaged on a motorized microscope (Nikon ECLIPSE NI-E). For α-cell mass analysis, we multiplied the average of glucagon-positive area:pancreas area ratio of five regions (200 μM apart) and pancreas weight. Analysis of area was done using NIH ImageJ software. See Table S1 for antibody dilutions information.

### Brain imaging and PVN quantification

Brains were fixed in 4% formaldehyde (PFA) and embedded in OCT compound. Embedded brains were sectioned into consequent 40-μM-thick coronal slices, starting at the dorsal edge of the hypothalamus and continuing to the ventral edge of the hypothalamus. Staining with primary (αCamKII or NeuN1) and secondary antibodies was conducted directly onto free-floating brain sections and placed onto charged microscope slides. Imaging of stained slides was performed on a motorized microscope (Nikon ECLIPSE NI-E). PVN Cre(+) GFP (in αOGT^KO^) and RFP (in i-αOGT^KO^) were conducted by counting number of CAG(+), NeuN1(+), and α-CaMKII(+) cells per three consequent sections of PVN, through use of NIH ImageJ software.

### Cell culture and quantitative PCR

αTC-1 (40) and βTC-6 (41) cells (a gift from Dr Meri Firpo, University of Minnesota), were maintained in Dulbecco's modified eagle medium (Thermo Fisher), with 15 mM HEPES, 0.1 mM NEAA, 10% FBS, 0.02% BSA, and penicillin/L–streptomycin. OSMI-1 was dissolved in DMSO prior to use. αTC-1 cells were pretreated with OSMI-1 at indicated concentrations for 8 or 24 h prior to collection. Cells were maintained in a humidified 37 °C, 5% CO_2_ incubator. RNA samples were prepared from three islet preparations and αTC-1 from each treatment group. Quantitative PCR was performed as previously described (42).

### Immunoprecipitation and western blot

αTC-1 and βTC-6 cells were sonicated for lysis in RIPA buffer (Cell Signaling Technology, CST) + 1% SDS (BioRad) + Protease and phosphatase inhibitors (CST). Following Pierce BCA protein quantitation (ThermoScientific), 500 μg of cell lysates was immunoprecipitated with either control IgG and FOXA2 (Abcam) or RL2 (Abcam) antibodies, followed by incubation with Protein A/G agarose beads (Pierce) as previously described (18). In total, 20 to 50 μg of cell lysates was resolved by sodium dodecyl (lauryl) sulfate-polyacrylamide gel electrophoresis, transferred to polyvinylidene fluoride membrane, blocked with 5% nonfat dry milk, and incubated with primary antibodies, prior to treatment with HRP-conjugated secondary antibodies. The blot was visualized with SuperSignal West Pico PLUS (ThermoScientific), per manufacturer’s protocol. Densitometry analysis was performed with NIH ImageJ software as previously described (16, 18).

### Islet isolation, *in vitro* glucagon-arginine secretion, and single-cell glucagon assay

We have previously described our islet isolation technique (43). For the *in vitro* glucose-inhibited glucagon secretion assay, 3 × 15 islet aliquots per mouse in Millicell culture inserts were preincubated in a high-glucose Krebs buffer (6 mM glucose) for 90 min before experimental 1-h incubations in 6 mM glucose followed by 1 mM glucose in Krebs (supernatants collected). After a 30-min rest at 6 mM (discarded), islets were finally incubated for 1 h in 1 mM glucose (collected)+ 20 mM Arginine (1 mM glucose + Arg). Supernatants were assayed for glucagon ELISA. Islet screens were collected into RIPA buffer (CST) with protease inhibitor cocktail (CST) and assayed for DNA with Quant-iT Pico Green dsDNA Assay (Molecular Probes). Single-cell glucagon content was conducted with a starting group of 100 to 125 isolated islets, dispersed in 0.25% trypsin (Gibco), plated onto coverslips, and rested for 24 h. Individual GFP-positive islet cells were collected using a heat-pulled glass capillary, manipulated with a Sutter micromanipulator, under a fluorescence microscope (Olympus BX51 600× magnification). Five cells per mouse were combined and lysed in exactly 20 μl of RIPA buffer (+protease inhibitors) and assayed for glucagon content. Five cells were combined per mouse/genotype due to a minimum glucagon threshold requirement of the ELISA (Mercodia). After glucagon measurement *via* ELISA, each five-cell glucagon value was divided by 5 to yield average single-cell glucagon content.

### Statistical analysis

Data are presented as mean ± SEM and were analyzed using unpaired, two-tailed Student’s *t*-tests and one-way ANOVA. Multiple outcome data was assessed using repeated measures two-way ANOVA. Statistical analyses were performed in GraphPad Prism version 7 with a significance threshold of *p* < 0.05.

## Data availability

All data are contained within this article and provided as supporting data.

## Conflicts of interest

The authors have declared that no conflict of interest exists.
