# Supplementary material for: O-linked N-acetylglucosamine transferase (OGT) regulates pancreatic α-cell function in mice
Source: J Biol Chem. 2021 Jan 16;296:100297. doi: 10.1016/j.jbc.2021.100297 (PMC7949098; doi:10.1016/j.jbc.2021.100297)
Supplement: Supplemental Figures S1–S6 [file mmc1.pdf]

Figure S1

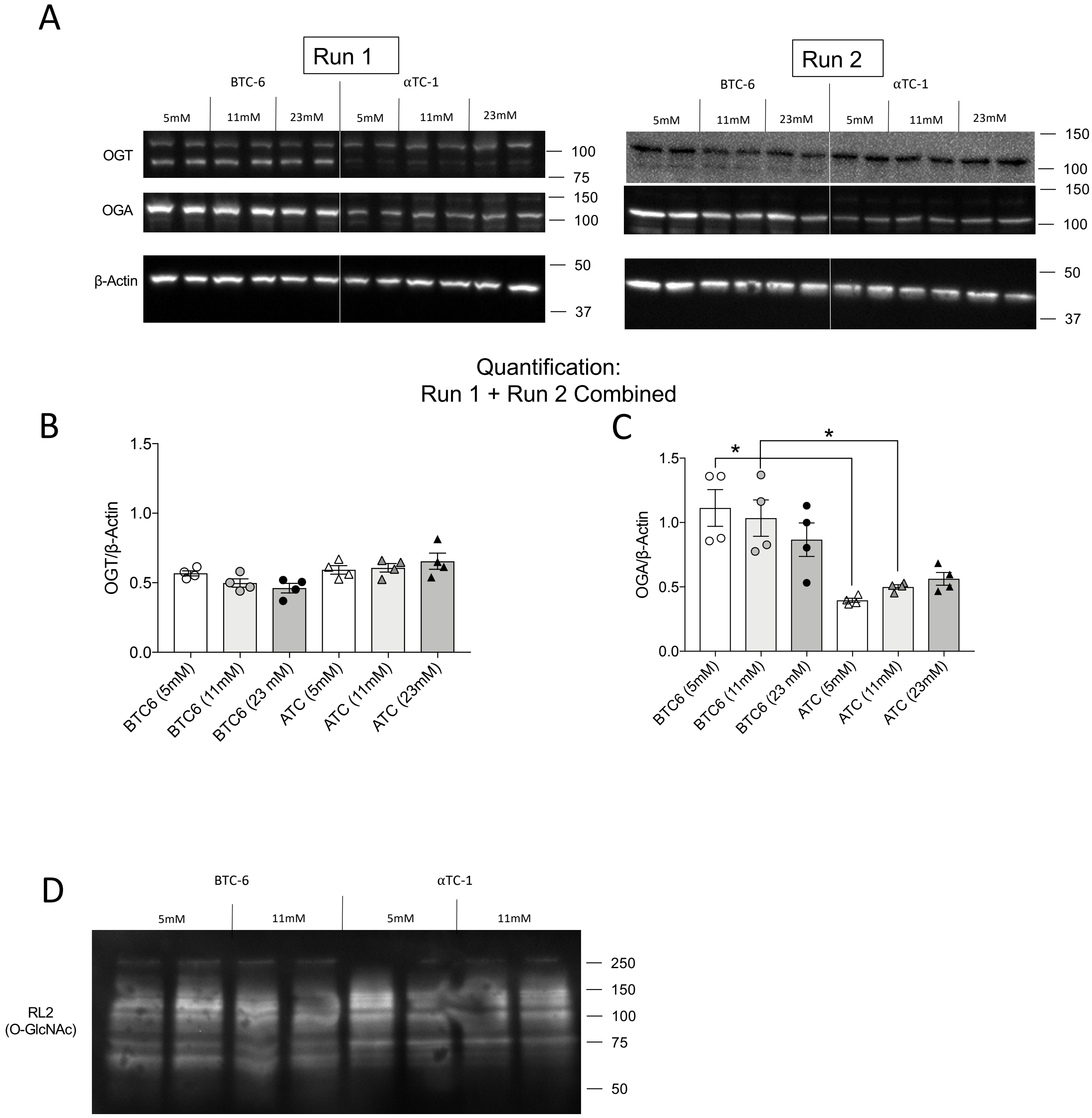

A
Run 1
BTC-6
αTC-1
5mM
11mM
23mM
OGT
OGA
β-Actin
100
75
150
50
37
Run 2
Quantification:
Run 1 + Run 2 Combined
B
OGT/β-Actin
1.5
1.0
0.5
0.0
BTC6 (5mM)
BTC6 (11mM)
BTC6 (23 mM)
ATC (5mM)
ATC (11mM)
ATC (23mM)
C
*
OGA/β-Actin
D
RL2
(O-GlcNAc)
250

Figure S2

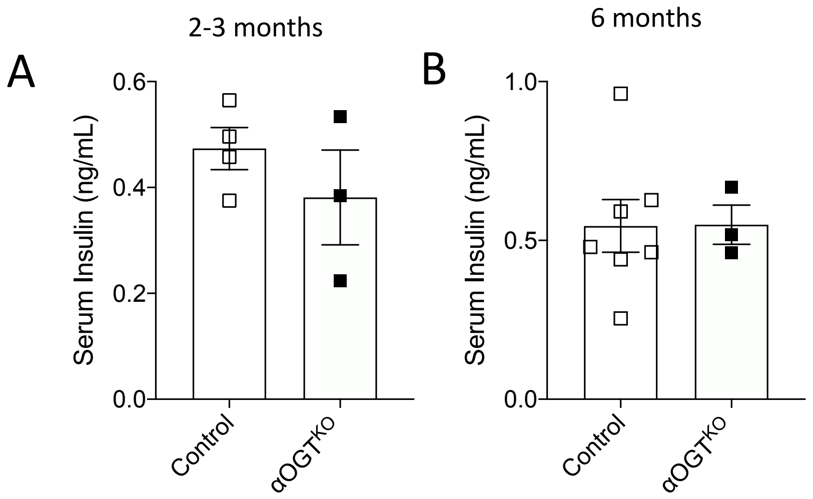

2-3 months
A
0.6
0.4
0.2
0.0
Serum Insulin (ng/mL)
Control
αOGTKO
6 months
B
1.0
0.5
0.0
Serum Insulin (ng/mL)
Control
αOGTKO

Figure S3

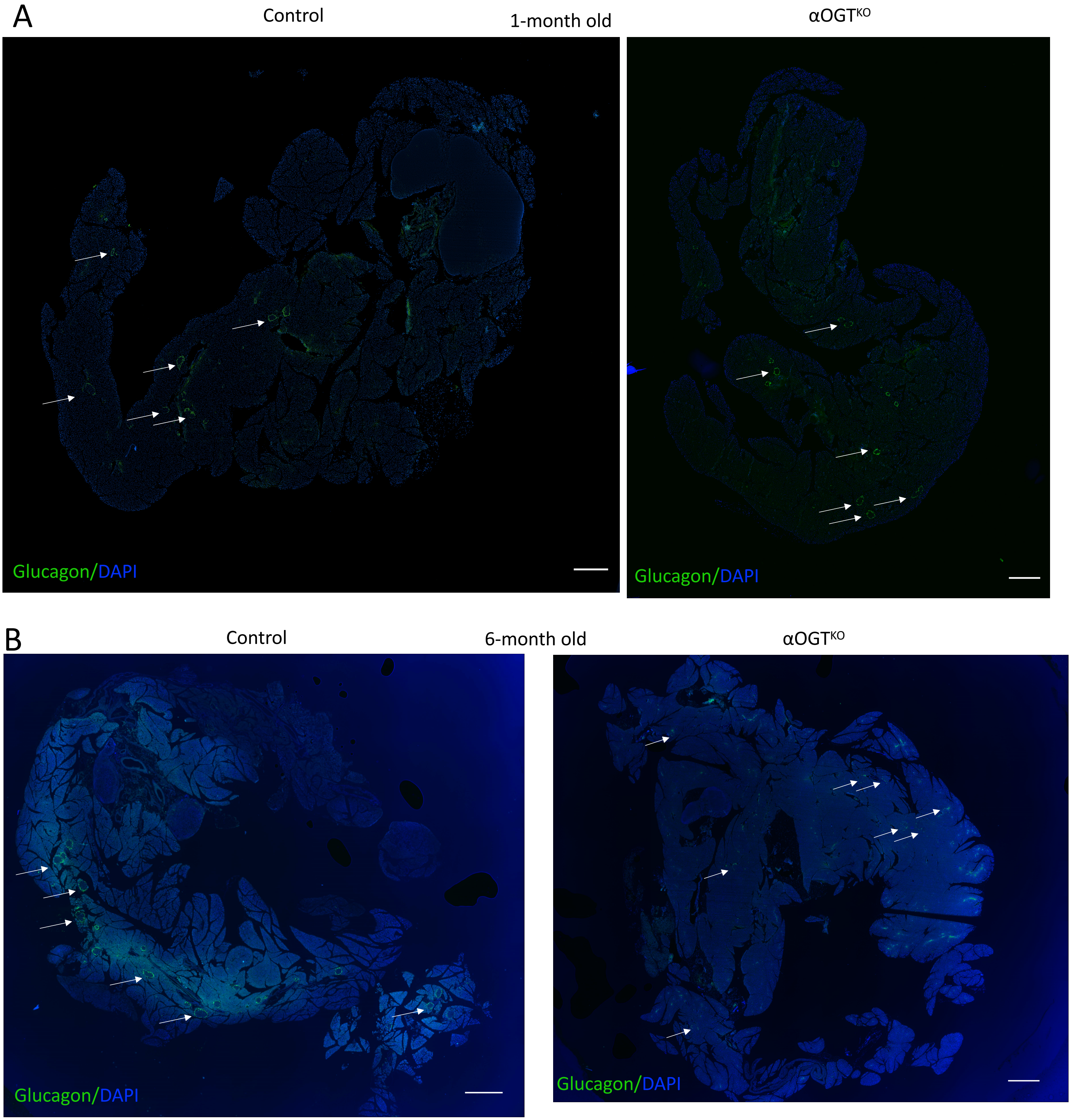
A
Control
1-month old
αOGTKO
Glucagon/DAPI
Glucagon/DAPI
B
Control
6-month old
αOGTKO
Glucagon/DAPI
Glucagon/DAPI

Figure S4

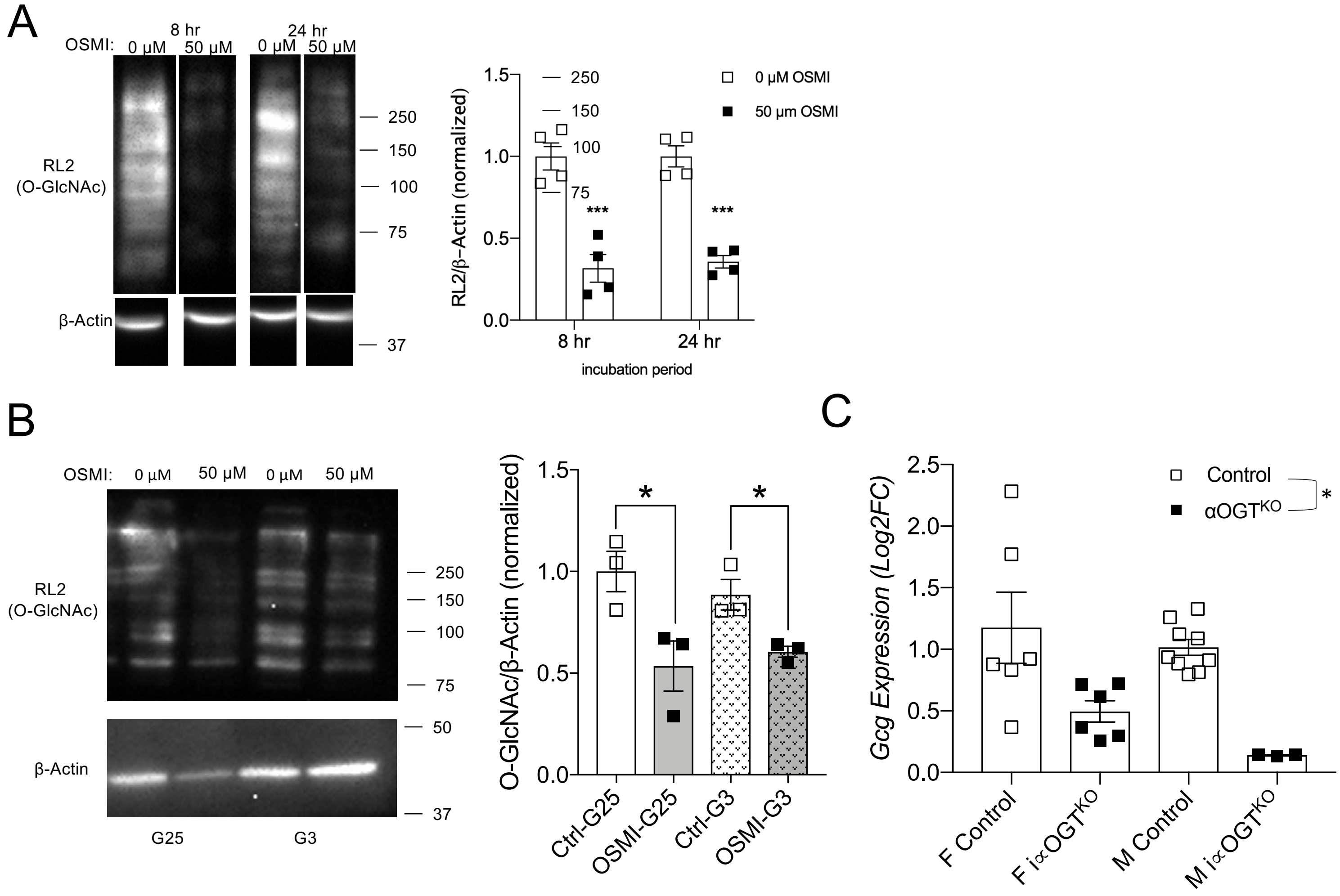
A
OSMI:
8 hr
24 hr
0 μM
50 μM
RL2
(O-GlcNAc)
β-Actin
250
150
100
75
37
RL2/β-Actin (normalized)
0 μM OSMI
50 μm OSMI
***
8 hr
24 hr
incubation period
B
OSMI:
0 μM
50 μM
RL2
(O-GlcNAc)
β-Actin
G25
G3
250
150
100
75
50
37
O-GlcNAc/β-Actin (normalized)
*
Ctrl-G25
OSMI-G25
Ctrl-G3
OSMI-G3
C
Gcg Expression (Log2FC)
Control
αOGT^KO
*
F Control
F i∝OGT^KO
M Control
M i∝OGT^KO

Figure S5

A

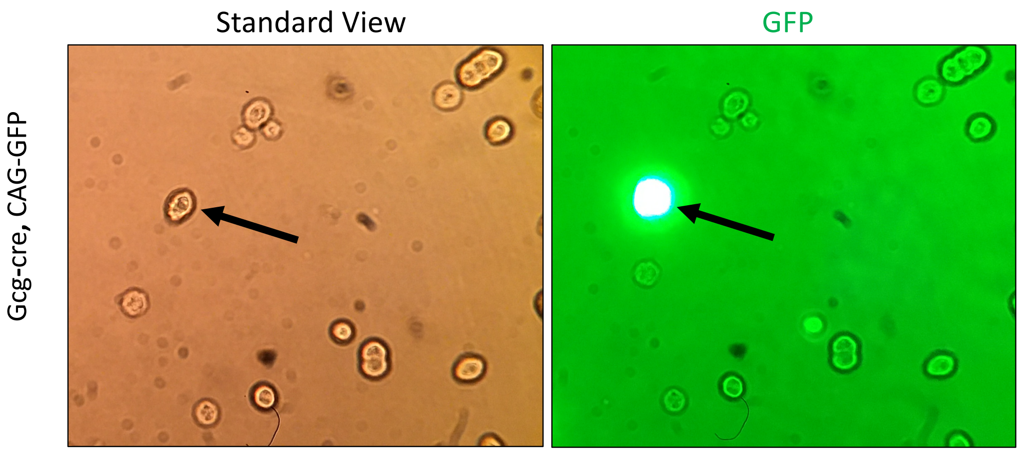

Standard View
GFP
Gcg-cre, CAG-GFP

B

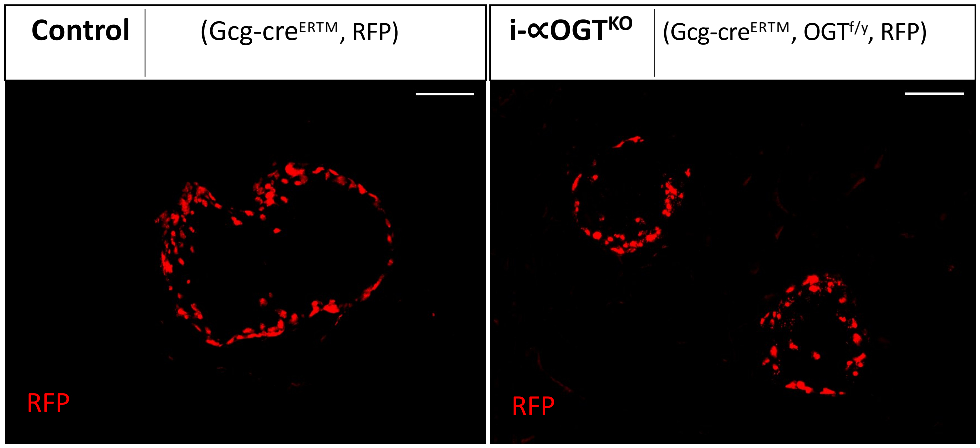

Control
(Gcg-creERTM, RFP)
i-∝OGTKO
(Gcg-creERTM, OGTf/y, RFP)
RFP
RFP

Figure S6

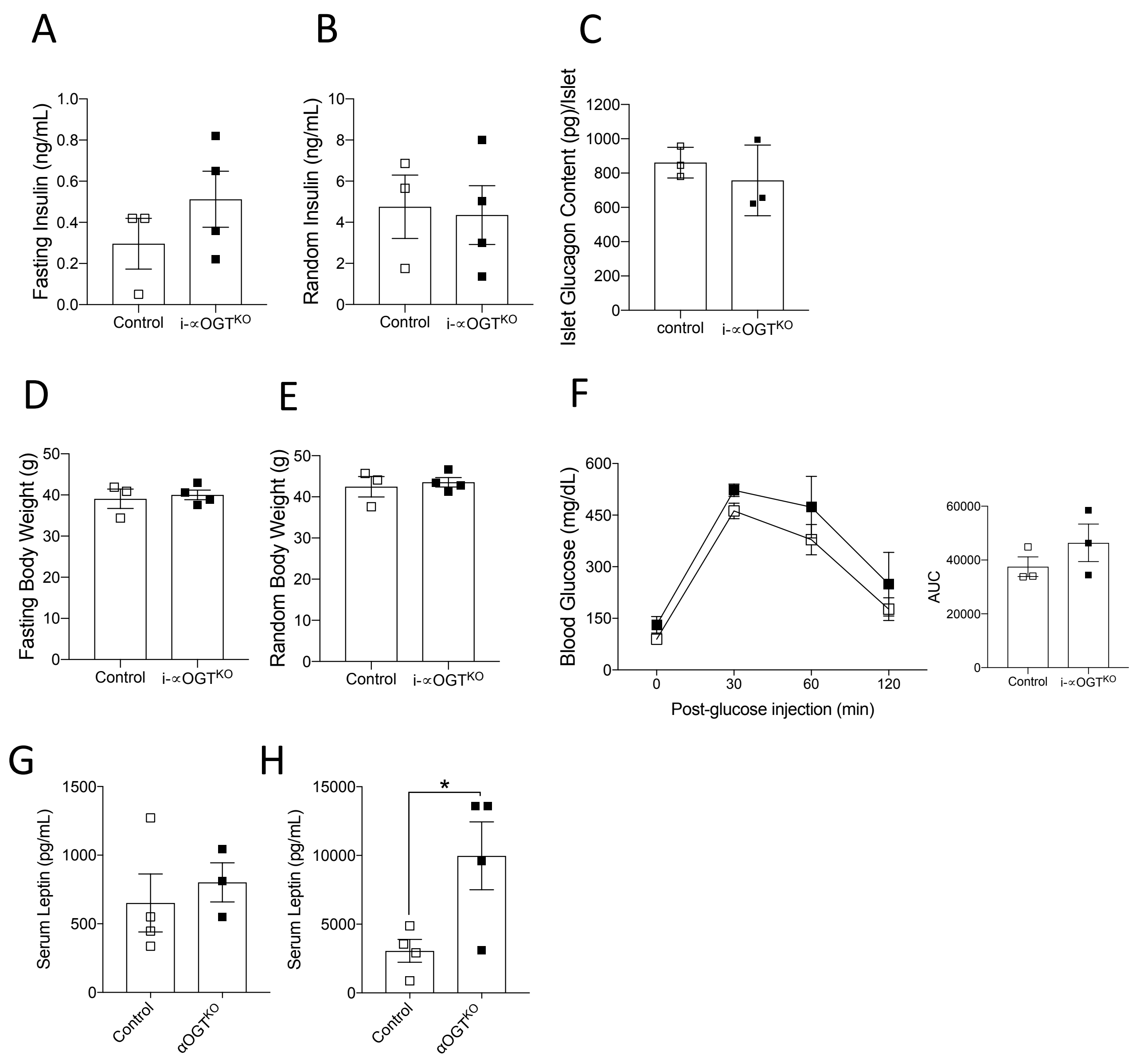

A
Fasting Insulin (ng/mL)
1.0
0.8
0.6
0.4
0.2
0.0
Control
i-∝OGT^KO
B
Random Insulin (ng/mL)
10
8
6
4
2
0
Control
i-∝OGT^KO
C
Islet Glucagon Content (pg)/Islet
1200
1000
800
600
400
200
0
control
i-∝OGT^KO
D
Fasting Body Weight (g)
50
40
30
20
10
0
Control
i-∝OGT^KO
E
Random Body Weight (g)
50
40
30
20
10
0
Control
i-∝OGT^KO
F
Blood Glucose (mg/dL)
600
450
300
150
0
0
30
60
120
Post-glucose injection (min)
AUC
60000
40000
20000
0
Control
i-∝OGT^KO
G
Serum Leptin (pg/mL)
1500
1000
500
0
Control
αOGT^KO
H
Serum Leptin (pg/mL)
15000
10000
5000
0
*
Control
αOGT^KO

Supporting Information Legends

Figure S1. OGT protein level and activity in α-cells and β-cells. Baseline levels of OGT and OGA protein (**A**) in αTC-1 and βTC-6 cells from varying glucose concentrations (5, 11 and 23mM), assessed by western blot, and quantified and normalized to β-Actin (**B, C**). Levels of total O-GlcNAcylation (RL2) in αTC-1 and βTC-6 cells grown in 5 or 11mM glucose for 24 hour assessed by western blot (**D**). Data represent mean ±SEM, n=4 per group, *p<0.05, comparing between groups. Analysis was done by unpaired, two-tailed Student's t-tests.

Figure S2. Serum Insulin level of constitutive αOGT^{KO} mice. Serum insulin level from young (2-3 months old; **A**) and old (6-months old; **B**) from control and αOGT^{KO} male mice (n=3-7).

Figure S3. Pancreas of αOGT^{KO} mice.
Representative images (4x magnification) of whole pancreas of αOGT^{KO} and control mice at 1 month (**A**) or 6 months (**B**) of age; Glucagon (Green) and DAPI (Blue). Scale shown is 500 µm.

Figure S4. Validation of OSMI-1, an OGT inhibitor, in αTC-1 cells.
Levels of O-GlcNAcylation in αTC-1 cells (**A,** n=4) after 8- or 24-hour treatment or in different glucose concentrations (3 and 25 mM) for 24-hours (**B**, n=3) with either OSMI-1 inhibitor (50 µM) or DMSO (control), assessed by western blot, and quantified and normalized to β-Actin. qPCR of glucagon transcript from male and female control and i-αOGT^{KO}, triplicate from each sample are presented (n=1-3 Male, n=2 Female). *p<0.05, comparing between groups. Analysis was done by unpaired, two-tailed Student's t-tests and 1-way ANOVA.

Figure S5. Presence of *Cre* activity in αOGT^{KO} and i-αOGT^{KO} mice.
(**A**) Dispersed islet cells from a Gcg-cre(+) mouse with *CAG*-GFP reporter, under bright field (left) and immunofluorescent (right) visualization. Microscope setting was 40x, and image was taken by an Apple iPhone, actual scale not available. (**B**) Immunofluorescent visualization of RFP in Gcg-cre^{ERTM} mice tagged with *CAG*-RFP cre reporter, at 15 weeks post-tamoxifen. 10x magnification image of a control (Gcg-creERTM, OGT^{WT}, RFP) mouse on the left side, and of an i-αOGT^{KO} (Gcg-Cre^{ERTM}, $OGT^{f/f}$, RFP) mouse on the right side. Scale shown is 100 µm.

Figure S6. i-αOGT^{KO} mice phenotype and circulating leptin levels in αOGT^{KO}
Fasting (**A**) and random fed serum insulin (**B**), islet glucagon content, normalized to islet number (**C**), fasting (**D**) and random fed body weight (**E**), and glucose tolerance test (i.p. injected glucose) (**F**) from control and i-αOGT^{KO} mice (n=3-4). Circulating leptin levels from 2-3 months old (**G**) and 6 months old (**H**) male control and αOGT^{KO} mice (n=3-4).

SUPPLEMENTAL TABLE 1
Summary table of reagents and resources utilized through the duration of this study.
