# Supplementary material for: O-linked N-acetylglucosamine transferase (OGT) regulates pancreatic α-cell function in mice
Source: J Biol Chem. 2021 Jan 16;296:100297. doi: 10.1016/j.jbc.2021.100297 (PMC7949098; doi:10.1016/j.jbc.2021.100297)
Supplement: Supplemental Table S1 [file mmc2.docx]

**Supplemental Table 1**

| **REAGENT or RESOURCE** | **SOURCE** | **IDENTIFIER** |
| --- | --- | --- |
| **Antibodies** | | |
| OGT (D1D8Q) Antibody, rabbit monoclonal (WB 1:500-1000) | Cell Signaling Technology | Cat# 24083 |
| OGA Antibody, rabbit polyclonal (WB 1:500-1000) | Sigma-Aldrich (Cell Signaling Technology) | Cat# SAB4200311 |
| Anti-O-Linked N-Acetylglucosamine Antibody [RL2], mouse monoclonal (WB 1:500-1000, IF: 1:500) | Abcam | Cat# ab2739 |
| Insulin Antibody, guinea pig polyclonal (1:400) | Dako | Cat# A056401-2 |
| Glucagon Antibody [K79bB10], mouse monoclonal (1:400) | Abcam | Cat# ab10988 |
| Recombinant Anti-CaMKII antibody [EP1829Y], rabbit monoclonal | Abcam | Cat# ab52476 |
| Anti-NeuN antibody, guinea pig polyclonal | EMD Millipore | Cat# ABN90 |
| Cleaved Caspase-3 (Asp175) Antibody, rabbit polyclonal | Cell Signaling Technology | Cat# 9661T |
| Cleaved PARP (Asp214) (D6X6X), rabbit monoclonal (Rodent Specific) | Cell Signaling Technology | Cat# 94885S |
| Beta-Actin (8H10D10) Antibody, mouse monoclonal (WB 1:1000-2000) | Cell Signaling Technology | Cat# 3700 |
| Sheep Anti-Mouse IgG (HRP-conjugate) (WB 1:10,000) | GE Healthcare | Cat# NA931 |
| Donkey Anti-Rabbit IgG (HRP-conjugate) (WB 1:15,000) | GE Healthcare | Cat# NA934 |
| Normal Mouse IgG | Santa Cruz Biotechnology | Cat# sc-2025 |
| **Chemicals, Peptides, and Recombinant Proteins** | | |
| Tamoxifen | Sigma | T5648 |
| Corn Oil | Sigma | C8267 |
| RPMI 1640 Media | Corning | 10-043-CV |
| HBSS (+ or - Ca2+, Mg2+) | Gibco | 14025-092 (+), 14175-095 (-) |
| Fetal Bovine Serum | GenClone | 25-514 |
| RIPA Buffer | Cell Signaling Technology | 9806S |
| Protease Inhibitor Cocktail | Cell Signaling Technology | 5871S |
| Phosphatase Inhibitor Cocktail | Cell Signaling Technology | 5870S |
| Collagenase P | Sigma-Aldrich (Roche) | 11213865001 |
| Pierce BCA Protein Assay Kit | ThermoFisher Scientific | 23227 |
| Humalog Insulin Lispro, injectable solution (100 iU/mL) | Eli Lilly and Company | NDC 0002-7510-01 |
| 50% Dextrose Injection, USP | Hospira, Inc. | NDC 0409-6648-02, RL-3040 |
| Sodium Pyruvate powder | Sigma | P5280 |
| Glucagon for Injection 1mg (1 unit) | Lilly USA | NDC 002-8031-01 |
| Albumin, Bovine Serum, Fraction V, RIA and ELISA Grade | Millipore Sigma | 126593 |
| DAPI mounting media | Vector Laboratories | H-1200 |
| RNAlater | ThermoFisher Scientific | AM7024 |
| DMSO | Sigma-Aldrich | D2650 |
| RestoreTM PLUS Western Blot Stripping Buffer | ThermoFisher Scientific | 46430 |
| **Critical Commercial Assays** | | |
| Mouse Ultrasensitive Insulin ELISA | ALPCO | 80-INSMU-E01 |
| Contour Blood Glucose Meter and Test Strips | Bayer | 9545C (meter), 06707202 (strips) |
| Mercodia Glucagon ELISA – 10ul | Mercodia | 10-1281-01 |
| Quantikine Glucagon ELISA | R&D Systems | DGCG0 |
| Quant-iT PicoGreen dsDNA Assay Kit | ThermoFisher Scientific | Ref: P11496 |
| Pierce BCA Protein Assay Kit | ThermoFisher Scientific | 23227 |
| SuperSignal West Pico PLUS Chemi | ThermoFisher Scientific | 34580 |
| **Experimental Models: Cell Lines** | | |
| βTC-6 | ATTC (Gift from Dr. Meri Fipro, UMN) | ATCC CRL-11506 |
| αTC-1 | ATTC (Gift from Dr. Meri Fipro, UMN) | ATCC CRL-2934 |
| **Experimental Models: Organisms/Strains** | | |
| Ogt Floxed Mouse - B6.129-Ogttm1Gwh/J | Jackson Labs | 004860 |
| Gcg-Cre Mouse – B6;129s-Gcgtm1.1(icre)Gkg/J | Jackson Labs | 030663 |
| Gcg-cre^ERTM^ Mouse – B6;129S-Gcgtm1.1(cre/ERT2)Gkg/J | Jackson Labs | 030681 |
| **Software and Algorithms** | | |
| ImageJ | https://imagej.nih.gov/ij/ | RRID:SCR_003070 |
| Prism v.7.0d | https://www.graphpad.com/scientific-software/prism/ | RRID:SCR_002798 |
| MyAssays | https://www.myassays.com/ | RRID:SCR_016562 |
